# Correlation between choline kinase alpha expression and ^11^C-choline accumulation in breast cancer using positron emission tomography/computed tomography: a retrospective study

**DOI:** 10.1038/s41598-023-44542-4

**Published:** 2023-10-17

**Authors:** Akane Ozawa, Masako Iwasaki, Kota Yokoyama, Junichi Tsuchiya, Ryutaro Kawano, Hiroshi Nishihara, Ukihide Tateishi

**Affiliations:** 1 Department of Diagnostic Radiology and Nuclear Medicine, Tokyo Medical and Dental University, 1-5-45, Yushima, Bunkyo-Ku, Tokyo, 113-8519 Japan; 2 Genomics Unit, Keio Cancer Center, Keio University School of Medicine, Tokyo, Japan

**Keywords:** Breast cancer, Medical research

## Abstract

Choline kinase (CK) is reportedly overexpressed in various malignancies. Among its isoforms, CKα overexpression is presumably related to oncogenic change. Choline positron emission tomography (PET) is reportedly useful for detecting and evaluating therapy outcomes in malignancies. In this study, we investigated the correlation between CKα expression and ^11^C-choline accumulation in breast cancer cells. We also compared the CKα expression level with other pathological findings for investigating tumour activity. Fifty-six patients with breast cancer (mean age: 51 years) who underwent their first medical examination between May 2007 and December 2008 were enrolled. All the patients underwent ^11^C-choline PET/computed tomography imaging prior to surgery. The maximum standardised uptake value was recorded for evaluating ^11^C-choline accumulation. The intensity of CKα expression was classified using immunostaining. A significant correlation was observed between CKα expression and ^11^C-choline accumulation (*P* < 0.0001). A comparison of breast cancer mortality demonstrated that strong CKα expression was associated with a shorter survival time (*P* < 0.0001). ^11^C-choline accumulation was also negatively correlated with survival time (*P* < 0.0001). Tumours with strong CKα expression are reportedly highly active in breast cancer. A correlation was observed between CKα expression and ^11^C-choline accumulation, suggesting their role as prognostic indicators of breast cancer.

## Introduction

Choline kinase (CK) is an enzyme that phosphorylates choline and generates phosphocholine via the Kennedy pathway. CK is activated by mitogenic stimulation or oncogenic transformations, and its product, phosphocholine, reportedly promotes DNA synthesis^1^. The final product of this pathway is phosphatidylcholine, the main component of the plasma membrane. Thus, CK plays an important role in mitosis.

CK overexpression is observed in various malignancies, such as breast^2^, lung, prostate, and colon^3^ cancer. Correlations between CK activity, CK overexpression, and high histological tumour grades have been reported in breast cancer^4^. Two isoforms are present in the CK family: CKα and CKβ. CKα has two splicing variants. Among these two isoforms, CKα overexpression is presumably associated with oncogenic change^5^. Without CKα, cells do not display proper microtubular structures, resulting in apoptosis^6^. Cancer cell survival presumably depends on the higher CKα levels compared with those of CKβ^6^. MN58b, a selective inhibitor of CKα, possesses antitumour activity against human breast cancer xenografts^2, 5^. CKα inhibitors also work against the survival of malignant B cells^7^. Therefore, CK is considered an important target for cancer therapy.

Increased choline levels have been reported in malignancies of several organs, such as the breast^8^, brain^9^, and prostate^10^, using magnetic resonance spectroscopy. ^11^C-choline positron emission tomography (PET), which is used for detecting and evaluating therapy in malignancies, has been reportedly used for brain^11^, prostate^12^, and breast^13, 14^ examinations. The sensitivity of ^11^C-choline PET/computed tomography (CT) is higher than that of magnetic resonance imaging for detecting lymph node metastasis in prostate cancer^15^. In breast cancer, ^11^C-choline PET has reliable reproducibility^13^, and its uptake is correlated with tumour aggressiveness^14^.

In a previous study, a correlation between mitosis and ^11^C-choline accumulation was reported. No similar relationship was observed between mitosis and ^18^F-FDG accumulation^16^. As mentioned above, CK, especially CKα, is considered to be an important factor in mitosis. We hypothesised that an association exists between the CKα level and ^11^C-choline uptake in breast cancer. In the present study, we investigated the correlation between CKα expression and ^11^C-choline accumulation in breast cancer cells. Long-term prognoses of the patients were evaluated, and the association between mortality and CKα expression and ^11^C-choline accumulation was investigated. We also compared CKα expression with other pathological findings to investigate tumour activity.

## Materials and methods

### Patients

In this study, 56 patients (mean age, 51 years; range 24–71 years) with breast cancer were enrolled. The patients underwent their first medical examinations between May 2007 and December 2008. All the patients underwent ^11^C-choline PET/CT. The diagnosis of breast cancer was confirmed by biopsy at least 1 month prior to the imaging studies. Patients with a performance status of 2 or more and those with multiple simultaneous malignancies were excluded. Hormone therapy was administered upon completion of all the imaging studies, and patients who had already received hormone therapy were excluded. Patients who underwent surgery after the imaging studies were included. Written informed consent was obtained from all the patients. This study was approved by the Institutional Review Board of the National Cancer Centre Hospital, Tokyo, Japan. This study complied with the guidelines of the Health Insurance Portability and Accountability Act.

### Phantom study

Imaging was performed using a whole-body PET/CT scanner (Aquiduo PCA-7000B; Toshiba Medical Systems, Tochigi, Japan). Prior to the study, a phantom study was conducted at two facilities to ensure imaging quality^17^. A NEMA phantom (NU 2-2001) was used for the study. The background radioactivity concentration was set to 2.6 ± 0.2 kBq/mL, which is close to the imaging conditions for clinical use. The radioactivity concentration of the hot portion was set four times greater than that of the background. Data were collected for 2–5 and 30 min in the dynamic and static acquisition modes, respectively. The data were visually assessed and used to evaluate the phantom noise equivalent count (NEC_phantom_), percentage contrast of the hot portion (Q_H10mm_), and percentage background variability (N_10 mm_). The reference values for the physical indices were as follows: NEC_phantom_ > 10.4 (counts), N_10mm_ < 6.2%, and Q_H10mm_/N_10mm_ > 1.9%. After the phantom study, the imaging conditions were set as follows: data acquisition, 180 s for one bed; field-of-view, 500 mm; iterations, 4; subsets, 14; matrix size, 128 × 128; filter, Gaussian 8 mm in full width at half maximum; and reconstruction, ordered subset expectation maximisation.

### Data acquisition

The synthesis of ^11^C-choline was based on the study by Hara et al.^18^. The patients fasted for at least 6 h before the imaging examinations. After urination, the patients were placed in a supine position with both arms raised. First, plain CT imaging was performed from the top of the head to the mid-thigh under free-breathing, at 120 KVp using an autoexposure control system (beam pitch, 0.875 or 1; and 1.5 or 2 mm × 16-row mode). Within 5 min of the intravenous injection of ^11^C-choline (average, 475.5 MBq; range, 457–491 MBq), PET imaging was performed for the patient's head to the mid-thigh.

### Image interpretation

CT, PET, and composite images were reviewed using proprietary software (Vox-base SP1000 workstation; J-MAC Systems, Sapporo, Japan). Two independent evaluators performed the visual and quantitative assessments. The findings were documented based on a consensus. The region of interest (ROI) was set as the contour area of increased uptake. If the uptake was heterogeneous, the ROI was set to cover the entire area. The standardised uptake value (SUV) was quantitatively recorded. The SUV_max_ was determined as the maximum accumulation within the ROI. Time-decay correction was not performed.

### Pathologic analysis

All patients underwent surgery. Surgical materials were fixed in 10% formalin and embedded in paraffin. Slices of 4 μm were prepared perpendicular to the long axis of the breast. Histological and nuclear grades were evaluated using the Elston–Ellis scoring system^19^. Oestrogen and progesterone receptor (ER and PgR, respectively) expression was evaluated using the H-scoring system described by McCarty et al.^20^. Human epidermal growth factor-2 (HER-2) immunostaining was performed using a 4B5 primary antibody. CKα immunostaining was performed using ab235938 antibody (ATLRAS 1–100; Atlas Antibodies AB; Sweden), and its expression was evaluated based on three grades from 0 to 2 (0, low intensity; 1, moderate intensity; and 2, high intensity). The following items were compared with CK α expression of invasive component, lymphatic invasion, histologic grade, nuclear grade, nuclear atypia, mitosis, extensive intraductal components, fat invasion, cutaneous invasion, muscular invasion, HER-2/neu, ER, and PgR.

### Statistical analysis

The patients’ medical records were evaluated until July 2022 to investigate their prognoses. Survival time was defined as the time from the date of the first medical examination to the last follow-up or death attributed to any cause. The correlations between CKα expression intensity and ^11^C-choline accumulation and other pathological findings were analysed using the chi-squared or Fisher's exact probability test. The correlation between CKα expression intensity, ^11^C-choline accumulation, and patient survival time was analysed using the log-rank test, and Kaplan–Meier curves were created. *P*-values were assessed using two-sided tests. A *P*-value of 0.05 or less was considered statistically significant.

### Ethical approval

This study was approved by the Institutional Review Board of the National Cancer Centre Hospital, Tokyo, Japan. This study complied with the guidelines of the Health Insurance Portability and Accountability Act.

### Consent to participate

Informed consent was obtained from all participants included in the study.

## Results

### Patients’ demographic data

The demographic data of all the patients are shown in Table 1. Between May 2007 and December 2008, 56 patients (mean age, 51 years; range 24–71 years) were enrolled. Tumours were located on the right and left sides in 29 (51%) and 27 (49%) patients, respectively. The mean tumour size was 28 mm (standard deviation [SD] ± 8.2; range 12–45). Among these tumours, 31 (55%), 8 (14%), 8 (14%), 5 (9%), and 4 (7%) were located in the lateral upper (C), medial upper (A), lateral lower (D), central (E), and medial lower (B) quadrants, respectively. A total of 48 patients (86%) had invasive tumours. Of these, 45 had no special type, 2 had micropapillary carcinoma, and 1 had mucinous carcinoma. Eight patients (14%) had non-invasive ductal carcinoma. We did not observe significant ^11^C-choline accumulation in lymph nodes. All patients were clinically free of lymph node metastases.

**Table 1:** Patient demographics.

Age (years)	51.0 ± 8.0 (24–71)
Tumour side	
Right	29 (51)
Left	27 (49)
Tumour size (mm)	28 ± 8.2 (12–45)
Main location	
Medial upper quadrant (A)	8 (14)
Medial lower quadrant (B)	4 (7.1)
Lateral upper quadrant (C)	31 (55)
Lateral lower quadrant (D)	8 (14)
Central (E)	5 (8.9)
Invasive tumour	48 (86)
Micropapillary	2 (4.2)
Mucinous	1 (2.1)
NST	45 (94)
Non-invasive tumour	8 (14)

Data are presented as mean ± standard deviation (range) or the number of cases. The data in parentheses are presented as percentages. *NST* No special type.

### Correlation between CKα expression and ^11^C-choline accumulation using PET

**Figure 1:**
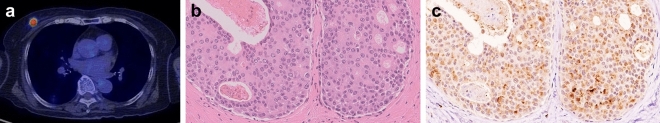
Imaging and pathologic findings of a 50-year-old woman. (**a**) ^11^C-choline positron emission tomography/computed tomography image. A focal accumulation was observed in the primary tumour (maximum standardised uptake value [SUV_max_] = 8.3). (**b**) Result of haematoxylin-eosin staining. A diagnosis of invasive ductal carcinoma was made. Both histological and nuclear grades were classified as 3. (**c**) Result of choline kinase alpha (CKα) immunostaining. High-intensity staining was observed.

**Figure 2:**
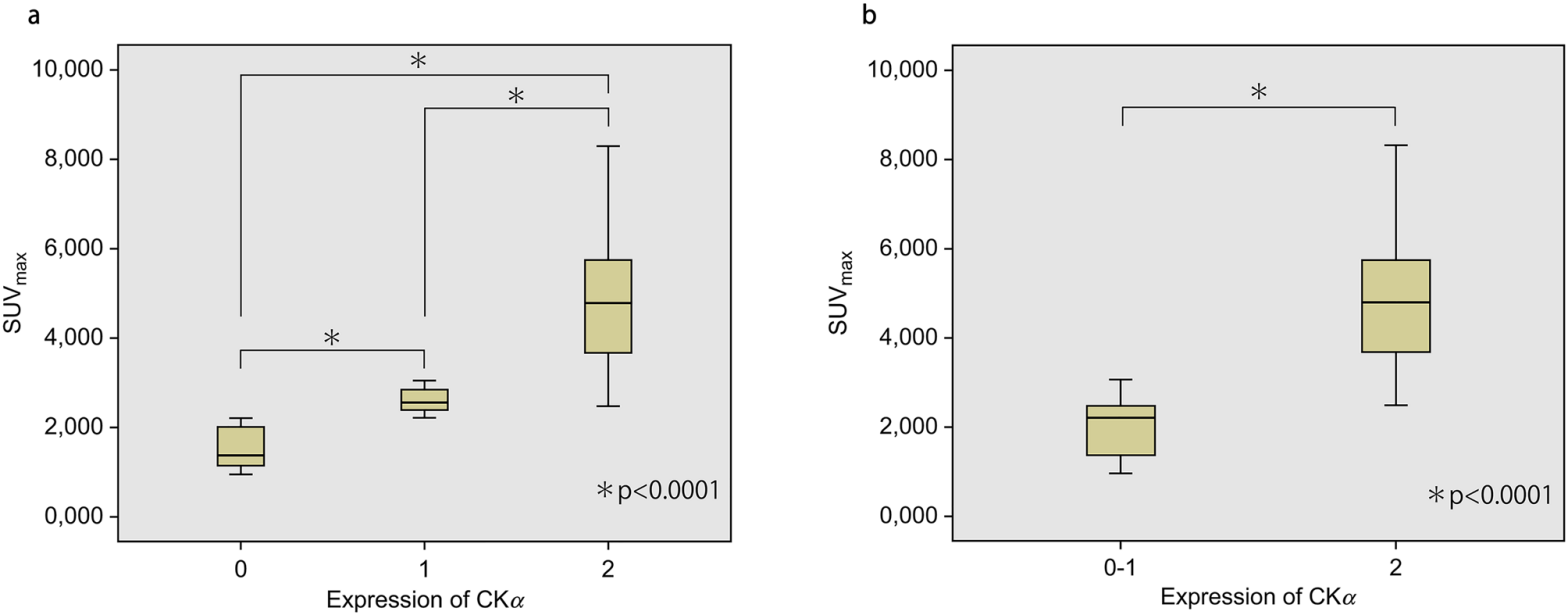
Correlation between CKα expression and ^11^C-choline accumulation using positron emission tomography. *CKα* Choline kinase alpha.

All the primary tumours were evaluated using ^11^C-choline PET/CT (Fig. 1). The mean SUV_max_ of all the cases was 3.2 (SD ± 1.8; range 0.95–8.3). After immunostaining for CKα, 18 (32%), 16 (29%), and 22 (39%) tumours were categorised as grades 0, 1, and 2, respectively. The correlation between the strength of CKα expression and SUV_max_ of ^11^C-choline PET is shown in Tables 2 and 3. The box plots are shown in Fig. 2a and b. A significant correlation was observed between these two variables (*P* < 0.0001). The correlation persisted upon comparing the weak (CKα expression graded as 0 or 1) and strong expression (CKα expression graded as 2) groups.

**Table 2:** Correlation between CKα expression and ^11^C-choline accumulation using PET.

	CKα expression			*P*-value
	0	1	2	
SUV_max_	1.5 ± 0.44 (0.95–2.2)	2.6 ± 0.26 (2.2–3.1)	4.9 ± 1.5 (2.5–8.3)	
SUV_max_ ≤ 3	18 (32)	15 (27)	2 (3.6)	< 0.0001
SUV_max_ > 3	0 (0)	1 (1.8)	20 (36)	

*SUV*_*ma*_, Maximum standardized uptake value; *PET* Positron emission tomography; *CKα* Choline kinase alpha.

**Table 3:** Correlation between CKα expression and ^11^C-choline accumulation using PET (CKα 0–1 vs. 2).

	CKα expression		*P*-value
	0–1	2	
SUV_max_	2.0 ± 0.66 (0.95–3.1)	4.90 ± 1.5 (2.5–8.3)	
SUV_max_ ≤ 3	33 (59)	2 (3.6)	< 0.0001
SUV_max_ > 3	1 (1.8)	20 (36)	

Data are presented as mean ± standard deviation (range) or the number of cases. The data in parentheses are presented as percentages.

*SUV*_*max*_ Maximum standardized uptake value; *PET* Positron emission tomography; *CKα* Choline kinase alpha.

### Correlation between CKα expression and ^11^C-choline accumulation and mortality

**Figure 3:**
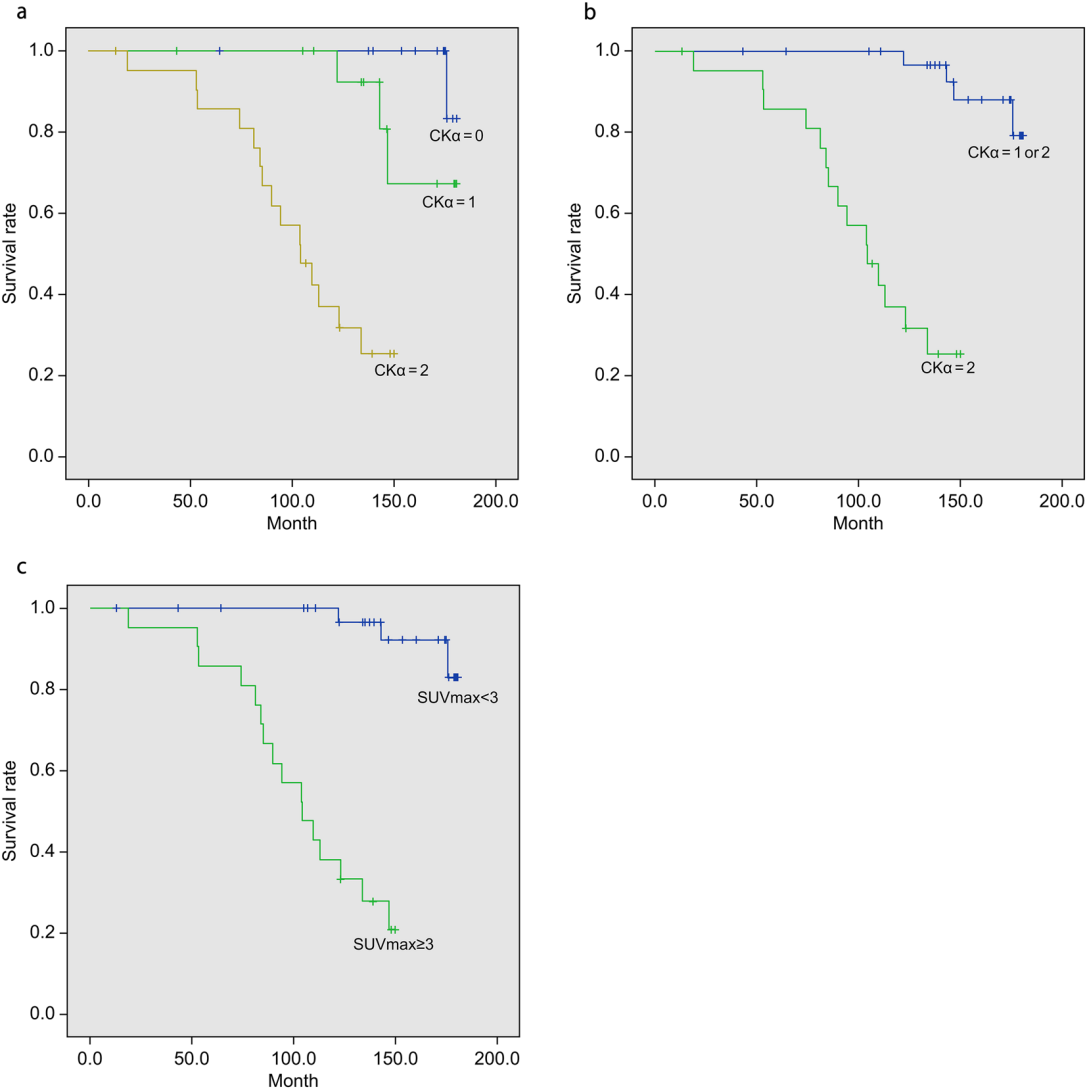
Correlation between CKα expression and ^11^C-choline accumulation and mortality. *CKα* Choline kinase alpha.

The patients’ medical records were evaluated until July 2022. During this period, 19 deaths owing to breast cancer were confirmed. Among these 19 cases, the mean survival time was 101 months (SD ± 36; range 18.7–176). The correlation between the strength of CKα expression and breast cancer mortality is shown in Tables 4 and 5. The Kaplan–Meier curves are shown in Fig. 3a and b. A significant difference was observed in the survival time based on the expression intensity of CKα (*P* < 0.0001). Survival time also significantly differed between the high-SUV (SUV_max_ > 3) and low-SUV (SUV_max_ ≤ 3) groups (*P* < 0.0001) (Table 6 and Fig. 3c).

**Table 4:** Correlation between CKα expression and mortality.

	CKα expression			*P*-value
	0	1	2	
Number of cases	18	16	22	
Number of death (%)	1 (5.6)	3 (19)	15 (68)	
Mean survival time (month)	180 ± 0.79	167 ± 6.5	104 ± 8.0	< 0.0001

*CKα* Choline kinase alpha.

**Table 5:** Correlation between CKα expression (CKα 0–1 vs. 2) and mortality.

	CKα expression		*P*-value
	0–1	2	
Number of cases	34	22	
Number of death (%)	4 (12)	15 (68)	
Mean survival time (month)	175 ± 2.8	104 ± 8.0	< 0.0001

*CKα* Choline kinase alpha.

**Table 6:** Correlation between ^11^C-choline accumulation and mortality.

	SUV_max_		*P*-value
	≤ 3	> 3	
Number of cases	35	21	
Number of death (%)	3 (8.6)	16 (76)	
Median survival time (month)		104 ± 11	
Mean survival time (month)	177 ± 2.5	105 ± 8.0	< 0.0001

Data are presented as mean ± standard deviation or the number of cases. The data in parentheses are presented as percentages.

*SUV*_*max*_ Maximum standardized uptake value.

### Correlation between the pathological findings and CKα expression

The correlations between the pathological findings and CKα expression strength are listed in Tables 7 and 8. Significant correlations were observed between CKα expression and the invasive components, mitosis, extensive intraductal components, fat invasion, ER, and PgR. Comparison of the weak and strong expression groups revealed significant differences in the invasive components, mitosis, extensive intraductal components, fat invasion, and PgR. No correlation was observed with ER.

**Table 7:** Correlation between the pathological findings and CKα expression.

	CKα expression			*P*-value
	0	1	2	
Invasive component				
Present	11 (20)	15 (27)	22 (39)	0.001
Absent	7 (13)	1 (1.8)	0 (0)	
Lymphatic invasion				
Present	13 (23)	9 (16)	12 (21)	0.48
Absent	5 (8.9)	7 (13)	10 (18)	
Histologic grade				
1	2 (3.6)	1 (1.8)	0 (0)	0.60
2	8 (14)	9 (16)	12 (21)	
3	8 (14)	6 (11)	10 (18)	
Nuclear grade				
1	5 (8.9)	6 (11)	4 (7.1)	0.78
2	5 (8.9)	4 (7.1)	7 (13)	
3	8 (14)	6 (11)	11 (20)	
Nuclear atypia				
1	1 (1.8)	1 (1.8)	0 (0)	0.71
2	9 (16)	9 (16)	10 (18)	
3	8 (14)	6 (11)	12 (21)	
Mitosis				
1	8 (14)	12 (21)	10 (18)	< 0.0001
2	10 (18)	1 (1.8)	0 (0)	
3	0 (0)	3 (5.4)	12 (21)	
Extensive intraductal components				
Present	9 (16)	3 (5.4)	2 (3.6)	0.01
Absent	9 (16)	13 (23)	20 (36)	
Necrosis				
Present	8 (14)	6 (11)	10 (18)	0.88
Absent	10 (18)	10 (18)	12 (21)	
Fat invasion				
Present	7 (13)	9 (16)	17 (30)	0.05
Absent	11 (20)	7 (13)	5 (8.9)	
Cutaneous invasion				
Present	1 (1.8)	2 (3.6)	2 (3.6)	0.78
Absent	17 (30)	14 (25)	20 (36)	
Muscular invasion				
Present	1 (1.8)	1 (1.8)	0 (0)	0.51
Absent	17 (30)	15 (27)	22 (39)	
HER-2 receptor				
0	7 (13)	6 (11)	11 (20)	0.12
1	10 (18)	4 (7.1)	7 (13)	
3	1 (1.8)	6 (11)	4 (7.1)	
Oestrogen receptor				
Positive	18 (32)	10 (18)	15 (27)	0.02
Negative	0 (0)	6 (11)	7 (13)	
Progesterone receptor				
Positive	18 (32)	11 (20)	11 (20)	0.002
Negative	0 (0)	5 (8.9)	11 (20)	

Data are presented as the number of cases. The data in parentheses are presented as percentages.

*HER-2* Human epidermal growth factor-2; *CKα* Choline kinase alpha.

**Table 8:** Correlation between the pathological findings and CKα expression (CKα 0–1 vs. 2).

	CKαexpression		*P*-value
	0–1	2	
Invasive component			
Present	26 (46)	22 (39)	0.01
Absent	8 (14)	0 (0)	
Lymphatic invasion			
Present	22 (39)	12 (21)	0.45
Absent	12 (21)	10 (18)	
Histologic grade			
1	3 (5.4)	0 (0)	0.36
2	17 (30)	12 (21)	
3	14 (25)	10 (18)	
Nuclear grade			
1	11 (20)	4 (7.1)	0.51
2	9 (16)	7 (13)	
3	14 (25)	11 (20)	
Nuclear atypia			
1	2 (3.6)	0 (0)	0.38
2	18 (32)	10 (18)	
3	14 (25)	12 (21)	
Mitosis			
1	20 (36)	10 (18)	< 0.0001
2	11 (20)	0 (0)	
3	3 (5.4)	12 (21)	
Extensive intraductal components			
Present	12 (21)	2 (3.6)	0.03
Absent	22 (39)	20 (36)	
Necrosis			
Present	14 (25)	10 (18)	0.75
Absent	20 (36)	12 (21)	
Fat invasion			
Present	16 (29)	17 (30)	0.03
Absent	18 (32)	5 (8.9)	
Cutaneous invasion			
Present	3 (5.4)	2 (3.6)	0.97
Absent	31 (55)	20 (36)	
Muscular invasion			
Present	2 (3.6)	0 (0)	0.25
Absent	32 (57)	22 (39)	
HER-2			
0	13 (23)	11 (20)	0.68
1	14 (25)	7 (13)	
3	7 (13)	4 (7.1)	
Oestrogen receptor			
Positive	28 (50)	15 (27)	0.22
Negative	6 (11)	7 (13)	
Progesterone receptor			
Positive	29 (52)	11 (20)	0.004
Negative	5 (8.9)	11 (20)	

Data are presented as the number of cases. The data in parentheses are presented as percentages.

*HER-2* Human epidermal growth factor-2; *CKα* Choline kinase alpha.

## Discussion

There have been several reports regarding ^11^C-choline accumulation in breast cancer. However, the relationship between the mechanism of ^11^C-choline accumulation and the pathological background had never been investigated. The present study demonstrated a correlation between CKα expression and ^11^C-choline accumulation using PET. Associations between ^18^F-fludeoxyglucose (FDG) and ^11^C-choline accumulation and the histological findings in breast cancer were demonstrated in a previous study^16^. Thus, mitosis correlated with ^11^C-choline accumulation. No similar relationship was observed between mitosis and ^18^F-FDG accumulation. As previously mentioned, CK overexpression promotes mitogenic progression^2^. This can be explained from a biochemical perspective.

Similar to that in breast cancer, a strong correlation between ^11^C-choline accumulation and CKα expression has been reported in prostate cancer^15^. CKα was suggested to be involved in choline metabolism in these malignancies. However, in gliomas, ^18^F-choline accumulation reportedly did not correlate with CKα expression^21^. Increased expression of mRNA and protein of CK in lung cancer has been reported; however, no correlation was observed between ^11^C-choline accumulation and these factors^22^. The small number of cases is one of the limitations of both studies. However, CKβ or other synthetic pathways may be predominant according to the type of malignancy.

We investigated the correlation between the strength of CKα expression and the pathological findings of breast cancer. As shown above, significant correlations were observed between CKα expression and the invasive components, mitosis, extensive intraductal components, and fat invasion. These findings suggest a relationship between CKα and breast cancer activity. According to a previous study^4^, CK enzymatic activity and overexpression, as measured using western blotting, correlate with the histologic grade. In the current study, CKα expression was examined using immunostaining, and no correlation was observed with the histological grade. Differences in the measurement methods could be attributed to this finding. The same study also demonstrated correlations among CK activity, overexpression, and ER deficiency. Although no association with ER was observed in the current study, an association between CKα expression and PgR deficiency has been suggested. This difference in hormone receptor expression may also arise from different measurement methods. We suggest that breast cancers with strong CKα expression tend to be hormone receptor-negative.

There are few reports comparing CKα expression and pathological findings to examine tumour activity in other malignancies. In the report on gliomas mentioned above, only one sample was strongly positive for CKα, and no significant relationship with tumour grade was observed^21^. According to a previous report on hepatocellular carcinoma, there was no relationship between CKα expression and tumour grade. However, CKα expression correlated with the cancer stage^23^. It is unclear whether the correlation between CKα expression and tumour activity is common among malignancies. Thus, we need further research.

In our present study, we found a significant correlation between CKα expression and survival time in breast cancer, suggesting that strong CKα expression was associated with a bad prognosis. A similar relationship between CKα and short survival time has been reported in hepatocellular carcinoma and non-small-cell lung cancer^23, 24^. A correlation between survival time and the SUV_max_ of ^11^C-choline was also observed in the present study. ^11^C-choline accumulation has been suggested to correlate with tumour aggressiveness^14^, and our results are consistent with this report. Both CKα expression and ^11^C-choline accumulation could be potential prognostic factors for breast cancer.

^18^F-FDG is the most widely used among PET formulations. A previous study targeting breast cancer reported that ^18^F-FDG accumulation had a significant correlation with both recurrence and mortality^25^. According to a meta-analysis, patients with high SUV_max_ of ^18^F-FDG in the primary lesion had a shorter event-free survival time. However, ^18^F-FDG accumulation has no significant correlation with overall survival^26^. A previous study targeting patients with bone metastases also reported that high ^18^F-FDG accumulation was associated with skeletal-related events and progression, but not with overall survival^27^. ^11^C-choline, which we used in the present study, might be more reflective of the mortality rate of breast cancer patients. However, the use of ^11^C-choline is less common than that of ^18^F-FDG. This is the first study that examined the correlation between ^11^C-choline accumulation and long-term prognosis in breast cancer, and further case study is required.

### Limitation

This study has some limitations. We analysed CKα expression in old surgical specimens. According to reports on the effects of slide ageing in breast cancer, the intensity of immunostaining for various proteins was lower in older specimens than in fresh ones^28, 29^. Despite this deterioration, associations between immunostaining results and other pathological parameters were maintained^28^. The intensity of immunostaining could have decreased owing to the ageing of the surgical materials. However, the association between CKα expression and the other findings should be maintained to some extent.

This study targeted primary tumours. Therefore, whether the associations between CKα expression, ^11^C-choline accumulation, and the other findings are maintained in recurrent or metastatic lesions is unclear. In addition, because this was an observational study, confounding factors were more likely to occur than in intervention studies. Although ^11^C-choline is a useful tracer for detecting malignant tumours, its short half-life makes it challenging in clinical practice.

## Conclusion

We suggest that strong CKα expression is associated with high tumour activity in breast cancer. A significant correlation was observed between CKα expression and ^11^C-choline accumulation. Both these factors may be considered prognostic factors for breast cancer.

## Data Availability

The datasets generated and/or analysed during the current study are available from the corresponding author upon reasonable request.
